# A high-density genetic map of *Schima superba* based on its chromosomal characteristics

**DOI:** 10.1186/s12870-019-1655-8

**Published:** 2019-01-25

**Authors:** Rui Zhang, Hanbo Yang, Zhichun Zhou, Bin Shen, Jijun Xiao, Bangshun Wang

**Affiliations:** 1 Research Institute of Subtropical Forestry, Chinese Academy of Forestry, Hangzhou, 311400 China; 2 Zhejiang Provincial Key Laboratory of Tree Breeding, Hangzhou, 311400 China; 3 Sichuan Academy of Forestry, Chengdu, 610081 China; 4 Longquan Academy of Forestry, Zhejiang, 323700 China

**Keywords:** Theaceae, *Schima superba*, Chromosome, Karyotype, Genotyping by sequencing (GBS), SNP, Linkage map, Growth traits, QTL

## Abstract

**Background:**

*Schima superba* (Theaceae) is a popular woody tree in China. The obscure chromosomal characters of this species are a limitation in the development of high-density genetic linkage maps, which are valuable resources for molecular breeding and functional genomics.

**Results:**

We determined the chromosome number and the karyotype of *S. superba* as 2n = 36 = 36 m, which is consistent with the tribe Schimeae (*n* = 18). A high-density genetic map was constructed using genotyping by sequencing (GBS). A F1 full-sib with 116 individuals and their parents (LC31 × JO32) were sequenced on the Illumina HiSeq™ platform. Overall, 343.3 Gb of raw data containing 1,191,933,474 paired-end reads were generated. Based on this, 99,966 polymorphic SNP markers were developed from the parents, and 2209 markers were mapped onto the integrated genetic linkage map after data filtering and SNP genotyping. The map spanned 2076.24 cM and was distributed among 18 linkage groups. The average marker interval was 0.94 cM. A total of 168 quantitative trait loci (QTLs) for 14 growth traits were identified.

**Conclusions:**

The chromosome number and karyotype of *S. superba* was 2n = 36 = 36 m and a linkage map with 2209 SNP markers was constructed to identify QTLs for growth traits. Our study provides a basis for molecular-assisted breeding and genomic studies, which will contribute towards the future research and genetic improvement of *S. superba.*

**Electronic supplementary material:**

The online version of this article (10.1186/s12870-019-1655-8) contains supplementary material, which is available to authorized users.

## Background

*Schima superba*, based on its original description, is formally placed in the tribe Schimeae (≡ Gordonieae) in Theaceae [1–3]. Theaceae is a family of subtropical and tropical trees in Asia containing approximately 17–20 genera and 500 species [1]. The genus *Schima* is closely related to the genera *Franklinia* and *Gordonia* and has approximately 20 species. *Schima* is an economically and ecologically important genus and is mainly distributed in southern China and the adjacent parts of East Asia, with 13 species (six endemic) present in China [1]. *Schima superba* is a typical large tree and dominant element in the subtropical evergreen broad-leaved forests of southern China. This species is valued commercially for its timber, and the wood is used for furniture and in construction. Additionally, these trees are used as fire breaks and thus help protect forests from fires [4–6].

*Schima* is distinct from other genera within Theaceae with regards to its chromosome number. The family Theaceae comprises three major tribes: Theeae, Schimeae, and Stewartieae. The tribe Stewartieae was the earliest to show differentiation at nearly 48 mya and has a chromosome number of *n* = 17. Theeae (*n* = 15) and Schimeae have a closer relationship, but their chromosome numbers are different. Previous studies indicate that all members of the tribe Theeae have the dominant basic chromosome number of *n* = 15 [3, 7, 8]. A chromosome number of 17 (*n* = 17) is the base number within the entire Stewartiae tribe [9]. In the tribe Schimeae, the basic chromosome numbers are either n = 15 or *n* = 18, and the count of n = 18 is more widely recognized in *Schima* than the original count number of n = 15 [2, 10–13]. The chromosome number of *S. superba* within *Schima* is still unclear, which limits studies on this species.

Due to the long (up to 30 years) breeding cycle of woody trees, there is an urgent need to improve upon traditional breeding methods [14]. Marker-assisted selection (MAS) is a useful tool for reducing the breeding cycle and has been used for many decades [15–18]. The development of a saturated genetic linkage map using molecular markers with high genome coverage is a prerequisite for the application of molecular plant breeding [19]. A molecular genetic linkage map is required as a fundamental resource for MAS; however, no high-density genetic maps have been constructed for *S. superba*. Therefore, an advanced breeding strategy and genetic maps are necessary for the identification of genes associated with important genotypes in *S. superba*. Genotyping by sequencing (GBS) has been used for the rapid development of thousands of segregating single nucleotide polymorphism (SNP) markers in large mapping populations at a low cost [20]. GBS has been widely used in high-density genetic linkage map construction [18, 21, 22] and quantitative trait locus (QTL) mapping in several plant species [23–26].

In the present study, we report the chromosome number of *S. superba* and describe its karyotype. We further performed hybridization between the individuals “LC31” and “JO32”. Based on the F1 full-sib, a high-density genetic map was constructed using the GBS approach. Phenotypic traits related to height, stem base diameter, growth rate, crown width, branching characters, and other growth-related parameters were mapped on this linkage map, and the associated SNPs were identified.

## Results

### Chromosome data

The mapping population consisted of 116 full-sibs derived from two selected trees from natural *S. superba* forests: LC31 (female parent, 50-years-old) and JO32 (male parent, 37-years-old). The parents exhibited differences in growth rate, woody yield, and wood quality. The hybridization was performed in 2013. A total of 116 full-sibs were harvested and planted on the forest farm of Longquan in 2014 and were used for the genetic map construction.

**Fig. 1:**
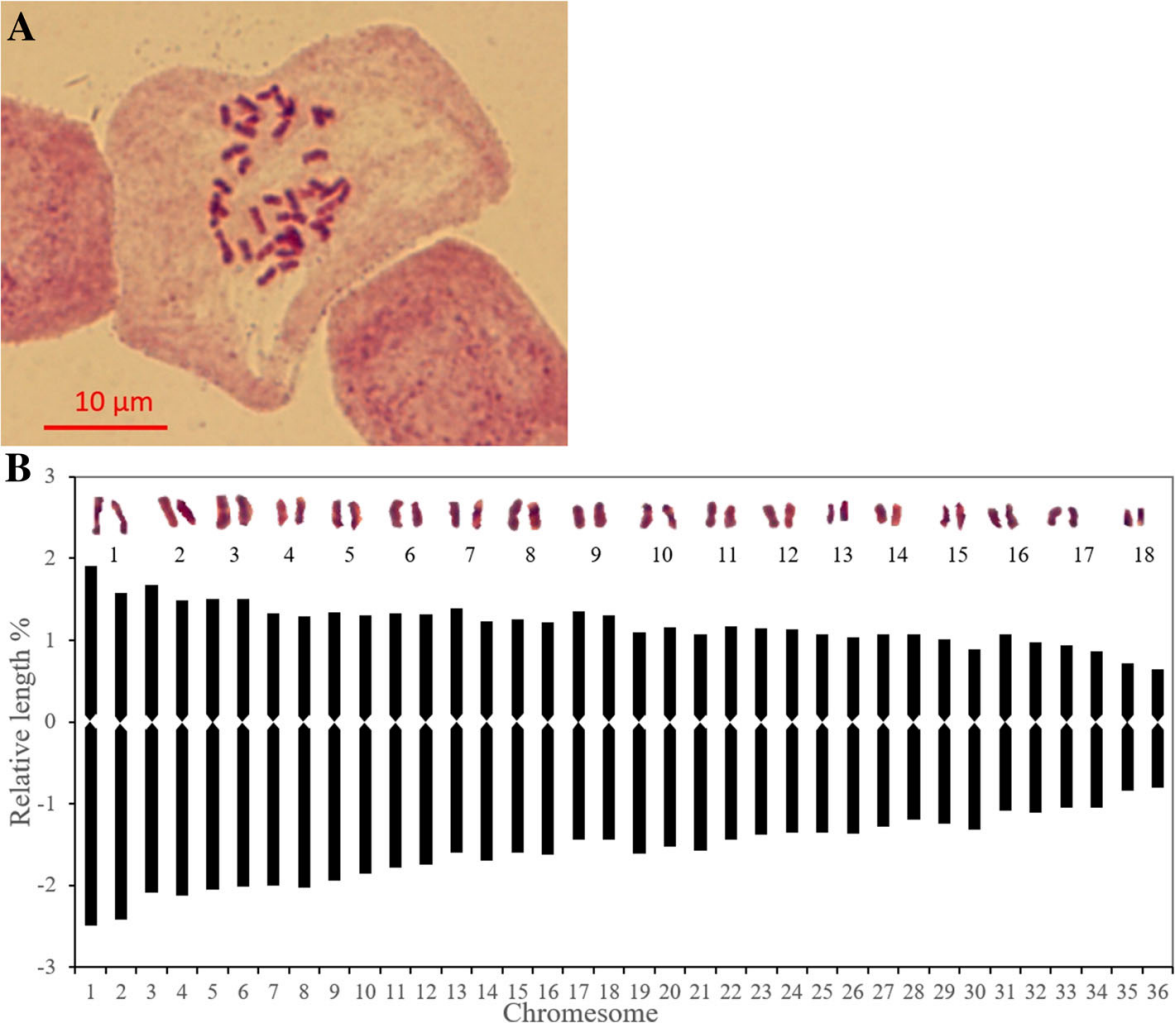
Cytological observations of *Schima superba*. **a** Metaphase, **b** Karyotype, formulated as 2n = 36 = 36 m

The mitotic metaphase indicated a chromosome number of 2n = 36 for *S. superba* (Fig. 1a). The absolute chromosome lengths ranged from 0.8 to 2.7 μm and were therefore classified as small chromosome types (Table 1). The chromosomes were determined to be 36 median (Fig. 1b; Table 1), which corresponded to a 3B karyotype symmetry. A secondary constriction was not observed. The arm ratios of most of the chromosomes were lower than 1.5 except one that was between 1.5 and 2.0 (Table 1) indicating low intra-chromosomal asymmetry.

**Table 1:** Mitotic metaphase chromosomes of *Schima superba*^a^

Number	Relative length %	Arm ratio	Centromere location type	Relative length index	The length type
1	2.45 + 1.74 = 4.19	1.41	m	1.53	L
2	2.11 + 1.58 = 3.69	1.33	m	1.34	L
3	2.03 + 1.50 = 3.53	1.35	m	1.29	L
4	2.02 + 1.31 = 3.33	1.54	m	1.22	M2
5	1.90 + 1.33 = 3.23	1.43	m	1.18	M2
6	1.77 + 1.32 = 3.09	1.34	m	1.13	M2
7	1.65 + 1.31 = 2.96	1.25	m	1.09	M2
8	1.61 + 1.24 = 2.85	1.30	m	1.04	M2
9	1.44 + 1.33 = 2.77	1.09	m	1.00	MI
10	1.57 + 1.13 = 2.70	1.40	m	0.97	MI
11	1.51 + 1.12 = 2.63	1.35	m	0.94	MI
12	1.36 + 1.14 = 2.50	1.19	m	0.89	MI
13	1.36 + 1.05 = 2.41	1.29	m	0.86	MI
14	1.24 + 1.07 = 2.31	1.15	m	0.81	MI
15	1.28 + 0.95 = 2.23	1.34	m	0.78	MI
16	1.10 + 1.02 = 2.12	1.07	m	0.74	S
17	1.05 + 0.90 = 1.95	1.16	m	0.68	S
18	0.82 + 0.68 = 1.50	1.21	m	0.50	S

^a^m, median; L, long chromosome; M2, medium long chromosome; MI, medium short chromosome; S, short chromosome

### Sequencing data quality assessment

The number of clean reads obtained from the female parent, male parent, and progeny were 37,926,886, 50,742,572, and 9,981,084–45,923,854 with an average of 20,550,577 clean reads. The 2.96 Gb of individual raw data were generated using a Hi-Seq platform for the parents and 116 progenies, yielding 343.3 Gb of high-quality sequences with an average Q20 ratio of 94.87%, a Q30 ratio of 87.83%, and a GC content of 35.17%. The average *Mse*I enzyme capture rate was 98.63%, validating the quality of the enzyme digest. No abnormal SNP calls were found, validating the genotyping accuracy.

In the absence of a suitable reference genome, a 137 Mb tag clustering by the male parent ‘JO32’ was used as the reference genome. A summary of the parental reads and reference genome alignment is shown in Table 2. For each progeny, the clean reads ranged from 9,981,084 to 45,923,854 bp, with an average of 20,550,577 bp (Additional file 1: Table S1). Moreover, the average mapping rate of the 116 full-sibs was 89.17%.

**Table 2:** Sequence depth and coverage statistics

Sample	Clean reads^a^	Mapped reads^b^	Mapping rate^c^	Average depth^d^	Coverage 1 × ^e^	Coverage 4 × ^f^
LC31	37,926,886	33,865,915	89.29%	41.50	80.12%	60.34%
JO32	50,742,572	49,233,750	97.03%	48.74	99.96%	90.55%

^a^ Number of reads used for the alignment

b Number of clean reads that mapped to the reference genome

c The percentage of reads that mapped to the genome

d Average sequencing depth

e Percentage of the reference genome with at least 1× coverage

f Percentage of the reference genome with at least 4× coverage

### GBS-based SNP identification

We used the GATK (type UnifiedGenotyper) software to determine 298,332 and 190,522 SNPs in the female and male parents, respectively. For F1 individuals, an average of 276,582 SNPs was discovered for an individual progeny. The heterozygosis SNP rate of the females and males was 72.04 and 98.86%, respectively. The progeny SNP results are provided in the Additional file 2: Table S2 File. To avoid false positive SNPs, the base number of the parent SNP was set as ≥4 and the base number of the progeny was ≥2.

**Fig. 2:**
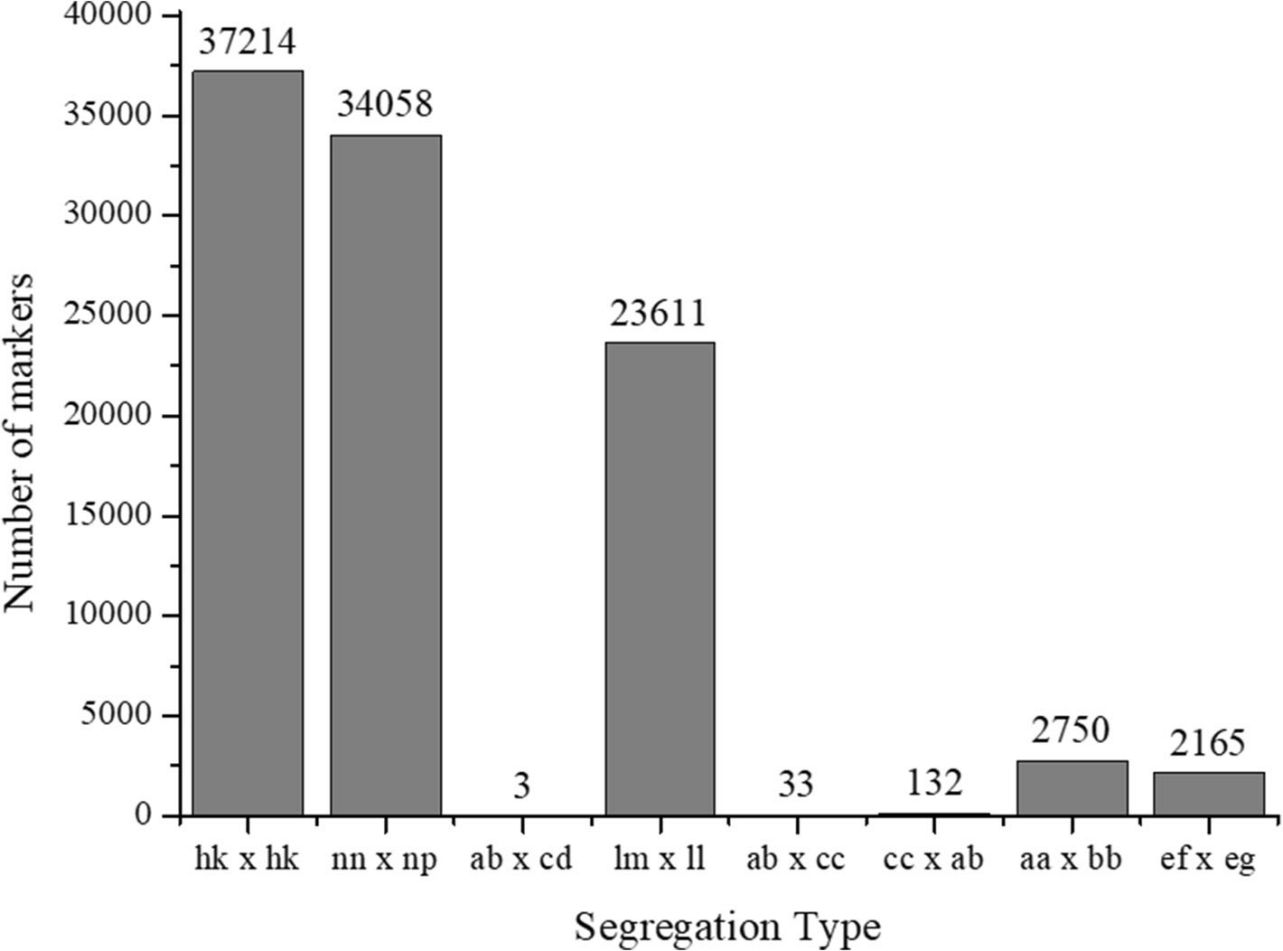
Segregation types of polymorphic SNP markers. The *x*-axis indicates the eight segregation types; the *y*-axis indicates the corresponding number of markers

A Bayesian model was used to detect 99,966 polymorphic loci (Fig. 2), which could be classified into eight segregation types according to the CP model in JoinMap 4.0. Among these, three major patterns including hk × hk, nn × np, and lm × ll accounted for nearly 94.92% of the loci, while the other five patterns, ab× cd, ab × cc, cc × ab, ef × eg, and aa × bb, accounted for only 5.08%. Thus, only segregation types hk × hk, nn × np, and lm × ll were selected for genotyping in F1 individuals, resulting in a total number of 94,883 polymorphic loci.

**Fig. 3:**
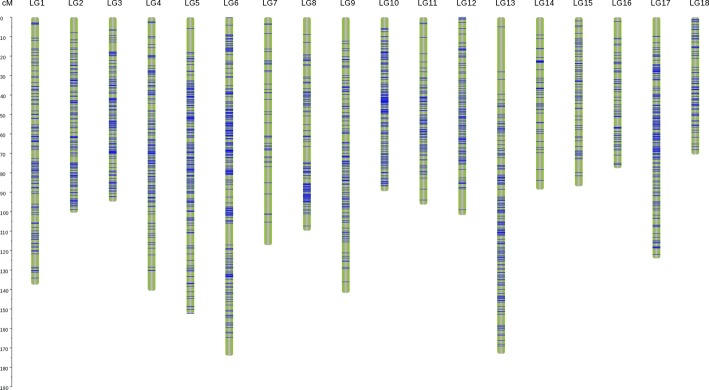
Genotyping-by-sequencing-based high-density genetic map of 116 full-sibs. The 2076.24 cM map included 2209 SNPs

### High-density genetic map development

A total of 28,856 markers were ultimately obtained after filtering the markers with complete coverage during genotyping. The available markers were filtered for < 65% integrity using a Chi-squared test with a threshold of *P* < 0.001. Ultimately, 519 markers with hk × hk, 639 markers with lm × ll, and 1051 markers with nn × np segregation types were used for map construction. Following data preparation, 1569 markers with types nn × np and hk × hk and 1137 markers with types lm × ll and hk × hk were used for the construction of male and female maps, respectively. On the male map, 1569 markers were placed into 18 LGs, and the genetic length was 1583.97 cM with an average marker interval distance of 1.01 cM (Additional file 3: Table S3). On the female map, 1137 markers were placed into 18 linkage groups (LGs), and the genetic length was 1459.19 cM with an average marker interval of 1.28 cM (Additional file 4: Table S4). The heat maps reflect the linkage relationships between the markers in a single linkage group (Additional file 5: Figure S1).

The two parent maps were then merged, and the integrated map spanned 2076.24 cM with 2209 markers placed into 18 LGs (Fig. 3). Among the 18 LGs, LG06 was the largest group with a genetic distance of 173.97 cM and 231 markers. LG18 was the smallest group with 84 markers, spanning 70.52 cM. The average marker interval ranged from 0.57 to 2.66 cM, with an average distance of 0.94 cM (Table 3). Between the markers, 2161 gaps (97.83%) were less than 5 cM, 40 gaps were between 5 and 10 cM, seven gaps were between 10 and 20 cM, and only one gap was larger than 20 cM, which was located on LG13.

**Table 3:** Genetic linkage group statistics of the integrated map

Linkage group	Number of SNP markers	Length (cM)^a^	Average distance (cM)^b^	Max. gap (cM)^c^
LG1	128	137.46	1.07	6.98
LG2	130	100.50	0.77	7.97
LG3	132	94.79	0.72	6.40
LG4	147	140.59	0.96	10.29
LG5	195	152.36	0.78	12.34
LG6	231	173.97	0.75	11.12
LG7	44	117.15	2.66	11.81
LG8	114	109.74	0.96	8.94
LG9	128	141.69	1.11	12.26
LG10	156	89.42	0.57	5.73
LG11	87	96.32	1.11	8.51
LG12	113	101.69	0.90	10.73
LG13	167	173.00	1.04	23.15
LG14	48	88.60	1.85	9.14
LG15	65	86.88	1.34	7.06
LG16	62	77.60	1.25	8.59
LG17	178	123.96	0.70	9.82
LG18	84	70.52	0.84	4.70
Average	123	115.35	0.94	–
Total	2209	2076.24	–	23.15

^a^Genetic distance of chromosomes (cM)

b Average genetic distance between markers (cM)

c Maximum gap between markers (cM)

### QTL mapping of growth traits

QTLs were mapped using the phenotypic data (Table 4) of 14 growth traits at an LOD (logarithmic) threshold of 3.0, from which 168 QTLs were identified (Table 5, Additional file 6: Table S5). These 14 traits could be classified into four categories namely I, II, III, and IV. Category I included the height characteristics: height of one-year-old seedlings (H1), height of three-year-old seedlings (H3), and height growth rate per year (HGR). The stem characteristics were designated in category II as stem base diameter of one-year-old-seedlings (SBD1), stem base diameter of three-year-old-seedlings (SBD3), and stem base diameter growth rate (SBDGR). The leaf characteristics leaf length (LL) and leaf width (LW) were classified in the third category (III). Crown width (CW), primary shoot number (PSN), maximum branching angle (MBA), maximum branching diameter (MBD), bifurcate trunk (BT), and height of bifurcate trunk (HBT) were classified in category IV for the branching characters. Thirty-four QTLs were identified for category I on chromosome LGs 1, 2, 3, 5, 8,10, 11, 12, 13, 16, and 17 with the percentage of phenotypic variation explained (PVE) by each QTL varying from 11.2 to 16.1%. The mean PVEs of the same traits were different in different years. Since the QTLs exerted the main- and side-effects, not all phenotypes were revealed at the same time. The mean PVEs of the QTLs were 12.1% for H1 and 14.1% for H3, and higher PVEs of the QTLs were located on LG13. The intervals of the five QTLs located on LG13 for HGR were 0.51, 22, 0.21, and 0.42 cM, respectively. The markers SsSNPLG13lm3242 and SsSNPLG13np4091 flanked the significant QTL qHGR-LG13. The two genotype calls AA and AG of SsSNPLG13lm3242 had an average HGR of 26 and 59%, respectively, and the two genotypes AA and AG of SsSNPLG13np4091 had an average HGR of 65 and 11%, respectively (Table 6).

**Table 4:** Variation analysis of phenotypic data of 14 growth traits^a^

Trait	Mean	Range	Coefficient of variation (%)
H1	43.32	15.00–71.00	26.65
H3	138.72	55.00–230.00	27.40
HGR	31.43	2.67–60.33	39.94
SBD1	4.76	2.12–6.90	22.77
SBD3	21.48	6.63–37.34	31.02
SBDGR	5.54	0.68–10.85	40.08
LL	11.88	8.00–18.00	14.27
LW	3.40	2.70–5.10	11.75
CW	99.03	32.50–165.00	26.60
PSN	9.74	3.00–22.00	37.54
MBA	76.04	45.00–100.00	18.32
MBD	9.71	3.50–20.48	33.82
BT	1.20	1.00–2.00	35.14
HBT	36.70	1.00–55.00	44.31

^a^ H1, the seedling height of the 1st year, cm; H3, the seedling height of the 3rd year, cm; HGR, height growth rate

per year, cm; SBD1, stem base diameter of 1 year, mm; SBD3, stem base diameter of 3 year, mm; SBDGR, SBD

growth rate per year, mm; LL, leave length, cm; LW, leave width, cm; CW, crown width, cm; PSN, primary shoot

numbers; MBA, maximum branching angle, °; MBD, maximum branching diameter, mm; BT, bifurcate trunk numbers; HBT, the height of bifurcate trunk, cm

**Table 5:** QTL identified for different growth traits

Traits	Number of QTLs	Linkage group	PVE^a^ (%)	LOD value
H1	17	2, 5, 8, 11, 12, 13, 16, 17	11.2–14.0	4.1
H3	7	1, 3, 10, 13	13.1–15.0	4.3
SBD1	5	13, 16, 17	11.2–12.7	4.3
SBD3	11	4, 6, 10, 17	12.9–17.9	4.2
HGR	10	1, 3, 8, 13	12.8–16.1	4.5
SBDGR	10	4, 6, 10, 17	12.8–18.2	4.3
LL	22	1, 3, 4, 5, 8, 17	11.2–16.0	8.6
LW	15	2, 11, 13, 17	11.3–13.6	4.1
CW	14	4, 13, 17	12.9–19.4	4.2
PSN	10	3, 4, 8, 17	12.8–22.8	4.5
MBA	15	2, 3, 4, 6, 10, 15, 18	13.0–17.7	4.2
MBD	5	1, 4, 10	12.8–14.2	4.3
BT	10	1, 5, 9, 12, 17	12.8–20.8	7.3
HBT	17	5, 6, 10, 12, 13, 17, 18	12.9–18.9	6.7

^a^Percentage of phenotypic variation explained by each QTL

**Table 6:** Summary of QTL for HGR, SBDGR, CW, PSN, MBA, BT and HBT traits identified in the full-sib populations of LC31 × JO32

Traits	QTL	Marker	Position	LOD	Genotype	Individual numbers	Mean value of the phenotype	LC31(♀)	JO32(♂)
HGR	qHGR–LG13–2	lm3242	LG13–121.31	3.78	AA	29	27.86	AG	AA
					AG	67	32.36		
					–	20	33.67		
	qHGR–LG13–4	np4091	LG13–143.52	3.85	AA	74	29.11	AA	AG
					AG	12	36.08		
					–	30	35.15		
SBDGR	qSBDGR–LG4–5	np2901	LG4–67.77	4.39	GG	1	5.08	TT	TG
					TG	28	6.51		
					TT	56	5.18		
					–	31	5.24		
CW	qCW–LG4–5	np2901	LG4–67.77	4.52	GG	1	92.50	TT	TG
					TG	28	114.14		
					TT	56	92.18		
					–	31	96.81		
	qCW–LG4–11	np2206	LG4–82.3	4.72	TA	21	115.69	TT	TA
					TT	55	91.86		
					–	40	100.06		
PSN	qPSN–LG17–3	lm2346	LG17–90.9	5.67	GA	53	8.56	GA	GG
					GG	59	10.69		
					–	4	10.75		
MBA	qMBA–LG10–2	np8947	LG10–27.44	4.26	GA	18	84.67	GG	GA
					GC	5	73.75		
					GG	58	75.20		
					AC	1	60.00		
					–	34	74.03		
BT	qBT–LG12–1	np7972	LG12–49.42	5.12	GG	69	0.10	GG	GA
					GA	42	0.16		
					–	5	0.00		
	qBT–LG17–2	np727	LG17–131.07	4.49	AA	51	0.20	AA	AG
					AG	48	0.05		
					–	17	0.07		
HBT	qHBT–LG17–1	hk3434	LG17–109.96	4.59	AG	47	27.00	AG	AG
					GG	44	27.50		
					AA	1	48.00		
					–	24	45.75		

For (II), 26 QTLs were identified on chromosome LGs 4, 6, 10, 13, 16, and 17 with PVEs varying from 11.2 to 18.2%. The mean PVEs of the QTLs were 11.9% for SBD1 and 14.2% for SBD3, and the QTL on LG4 had the highest PVE (18.2%) (Additional file 6: Table S5). The intervals of the six QTLs located on LG4 for SBD growth rate per year (SBDGR) were 13.21, 25.7, 0.03, 0.9, and 14.53 cM, respectively. The markers SsSNPLG4np2901 revealed the significant QTL qSBDGR-LG4. The genotype calls TG and TT had average SBDGRs of 25 and 49%, respectively (Table 6).

We identified 37 QTLs for category III on LGs 1, 2, 3, 4, 5, 8, 11, 13, and 17 with PVEs varying from 11.2 to 16.0%. Category IV had 71 QTLs that were identified on LGs 1, 2, 3, 4, 5, 6, 8, 9, 10, 12, 13, 15, 17, and 18 with PVEs varying from 12.8 to 22.8%. The QTL on the LG4 marker SsSNPLG4np2206 had the highest PVE (19.4%) for CW, and another marker SsSNPLG4np2901 (identified in SBDGR) had a higher PVE = 18.6%. The SsSNPLG17lm2346 marker had the highest PVE (22.8%) for PSN. The LG10 SsSNPLG10np8947 marker had the highest PVE (17.7%) for MBA; the LG12 SsSNPLG12mp7972 marker had the highest PVE (20.8%) for BT, the LG17 SsSNPLG17np727 marker had the highest PVE (18.5%) for BT, and the LG17 SsSNPLG17hk3434 had the highest PVE (18.9%) for HBT.

## Discussion

Morphological and karyotype analysis of *S. superba* indicated that it has a diploid chromosome number (*n* = 18) with three large, five medium-large, seven medium-small, and three small sized chromosomes with a median centromere (Fig. 1, Table 1). *Gordonia* and *Schima* are the two biggest genera in the tribe Schimeae (≡Gordonieae). *Gordonia* has the highest species count with *n* = 15 [2, 10, 27–29], and only one North American species (*G. lasianthus*) has a different chromosome number of *n* = 18 [30]. Yang (2004) indicated that *Gordonia* should be further classified into two genera: one being the Chinese *Gordonia* species with *n* = 15, which should be classified into *Polyspora* in tribe Theeae, whereas the other North American *Gordonia* (*G. lasianthus*) with n = 18 should form the monotypic genus *Gordonia* s.str [2]. Our results confirmed that the species chromosomal number in *Schima* is *n* = 18. Previous studies showed that *n* = 18 for most species in this genus [28, 29, 31, 32], and only *Schima wallichii* has *n* = 15 [33] or *n* = 18 [2, 34]. Bloembergen and Keng regarded the genus as monotypic and subdivided *S. wallichii* into nine or more geographically separated species [35, 36]. Our results corroborated the study of Wang (2006) where the base chromosome numbers in tribes Theeae, Schimeae (≡Gordonieae), and Stewartieae in Theaceae are *n* = 15, *n* = 18, and *n* = 17, respectively [3].

The construction of genetic linkage maps and the identification of growth trait-related QTLs will facilitate future genetic and breeding studies in *S. superba*. However, the absence of a genetic map has prevented the use of QTLs from this species in breeding programs. GBS is a rapid, efficient, and cost-effective strategy for SNP development, genetic linkage map construction, marker-based complex trait selection, and draft genome assembly in many species with or without reference genomes [18, 20, 37–40]. We generated 343.3 Gb of raw sequences from which 99,966 SNPs and 94,883 (94.9%) polymorphic SNPs were detected from the two parents and the 116 full sibs. The integrated genetic linkage map was divided into 18 LGs and comprised 2209 SNP markers, which spanned 2076.24 cM with an average marker interval of 0.94 cM. Accordingly, the high-quality SNP-based map will provide a basis for MAS and genomic studies, which should contribute to the genetic improvement of *S. superba*.

Plant morphological traits influence cultivation area, cultivation patterns, yields, and planting efficiency in forests. Woody plant traits such as higher individual height, thicker stem base diameter, fewer branches or bifurcate trunks, fast growth rate, higher wood density, wood durability, lower wood shrinkage and fissure, and greater wood strength are favored by forest geneticists and breeders [14, 41]. We have a good understanding of the effect of individual QTLs on phenotypes and their position in the genome [42]. However, QTLs discovered in *Pinus radiata* experimental populations explain only 0.78–3.8% of the variation in juvenile wood density and diameter [43]. We developed 168 QTLs from 14 growth traits, which varied from 11.2–22.8% (Table 4).

Plant growth and branching pattern traits are complex dynamic traits that are regulated by the interactions of many genes that may behave differently during different growth stages. Some chromosome segments may be associated with different traits at different growth periods, and QTLs can be detected throughout various stages of plant development. However, certain QTLs are conditional and are found only at specific growth stages [44, 45]. For example, the QTL affecting height located in the interval from 45.95–143.94 cM on LG13 and the QTL affecting SBD located in the interval from 21.21–94.78 cM on LG17 appeared to be unconditional and were detected in the measurements from years one and three (Additional file 6: Table S5). However, the minor QTL of height located at 92.17 cM at LG2 was found only at the one-year stage, and the QTL of height located at 69.55 cM at LG10 was found only at the three-year stage (Additional file 5: Table S5). Ten out of the 168 QTLs with LOD scores of 3.78–5.67 affecting seven growth traits were detected, and 15.8–22.8% of the phenotypic variance was explained by the QTLs (Table 5).

## Conclusion

We further examined the association between the actual segregation of the SNP markers closest to the QTL peaks and the traits of interest in the mapping population. The relationship between the genotypes of the linked markers and the average phenotypic values are displayed in Table 5. For SsSNPLG13lm3242 and SsSNPLG13np4091, the individuals harboring the homozygous AA genotype exhibited significantly lower HGR. The “G” allele, in this case, was strongly correlated with increased vertical height. However, the presence of “G” in the marker SsSNPLG4np2901 was associated with increased stem base diameter (SBD). Other markers, including SsSNPLG4np2901, SsSNPLG4np2206, and SsSNPLG10np8947, showed a “co-dominant” effect, where the average phenotypes of the heterozygous “TG,” “TA”, and “GA” individuals were higher than those of the homozygous groups. The genotypes of all of these SNPs for these QTLs in the full-sib were much more similar to the father line (JO32) and showed higher phenotype trait values (Table 5).

## Methods

### Morphological and karyotype characters

Seeds and seedlings of *S. superba* were collected from the Longquan region of Zhejiang province, China. The seeds were germinated in Petri dishes in a growth chamber, and the seedlings were cultivated in the nursery of the Longquan Forestry Academy.

Roots (1 cm) were removed from the seedlings or germinating seeds and pretreated with 8-hydroxy quinoline for 4 h at 4 °C, fixed for 24 h in Carnoy’s fluid (absolute alcohol: glacial acetic acid =3:1) at 4 °C, washed with 70% alcohol (*v*/v), washed with distilled water, macerated in 1 M hydrochloric acid at room temperature for 10 min and at 60 °C for 20 min, and then macerated in distilled water for 1 h. The root tips were then removed, compressed, and stained with carbol Fuchs. The cytological classification of the resting and mitotic prophase was performed as described by Tanaka [46]; the classification of karyotype symmetry was according to Stebbins [47]; and the use of symbols for the description of the chromosomes was according to Levan [48].

### Experimental population and phenotypic measurements

The mapping population consisted of 116 full-sibs derived from two select trees from natural *S. superba* forests: LC31 (female parent) and JO32 (male parent). The parents exhibited obvious differences in several phenotypes, such as growth rate, woody yield, and quality. They were twig-grafted in 2009 and kept in Longquan, Zhejiang, China (latitude: 28°03’N, longitude: 119°06′E, mean altitude: 200–300 m). The mean annual air temperature was 17.6 °C, and the rainfall was 1664.8–1706.2 mm. The hybridization was performed in 2013. A total of 116 full-sibs were harvested and planted on the forest farm of Longquan in 2014 and were used for the genetic map construction.

In November 2015, H1, SBD1, LL, and LW of one-year-old seedlings were measured, and in November of 2017, H3, SBD3, HGR, SBDGR, CW, PSN, MBA, MBD, BT, and HBT of three-year-old seedlings were measured. All measurements were used for the QTL analysis.

### DNA extraction

Young leaf samples were individually collected from the two parents and 116 full-sibs for DNA extraction. All samples were immediately frozen in liquid nitrogen and preserved at − 80 °C until extraction. The genomic DNA was extracted using a Plant Genomic DNA Isolation kit (Dingguo, Beijing, China) following the manufacturer’s instructions. The DNA purity and concentration were determined using a NanoDrop 1000 (Thermo Fisher Scientific, USA) and assessed on 1% agarose gels.

### GBS protocol and library construction

A GBS strategy (Novogene, Beijing, China) was used to develop the SNP markers. First, we performed a GBS pre-design for restriction enzyme selection. The GBS library was constructed by digesting the genomic DNA with a *Mse* I, *EcoR* I, and *Hae* III enzyme combination with subsequent ligation to barcodes, after which each sample was amplified in the multiplex PCR. The desired fragments were selected for library construction.

Next, we performed a standard analysis of the raw data. The Illumina HiSeq™ sequencing platform (Illumina, San Diego, CA, USA) was used for paired-end (PE) 150 sequencing. Then, based on the analysis of the original data, we conducted advanced analyses, and DNA library assembly was followed by HiSeq sequencing with the removal of reads with adapters, low-quality base calls, or uncalled bases.

Finally, in the progeny GBS-Seq analysis, we analyzed the number of reads cut by *Mse*I at both ends of each screened read, and the reads that did not contain these restriction sites were discarded. The specific reads and the ratio of the total number of reads to the number of enzyme-captured reads were also recorded. Then, Burrows-Wheeler Aligner (BWA) software [49] was used to align the clean reads against the reference genome (settings: mem -t 4 -k 32 -M -R). The reference genome was selected using a large amount of data from the male parent and was clustered and built to obtain a consistent sequence. The reads of the male parents, allowing up to six base mismatches, were clustered using the Stacks software [50, 51] and used to select the groups that contained read support numbers up to 3. These were clustered to obtain the final reference sequence. The alignment files were converted to bam files using SAMtools software [52]. If multiple read pairs had identical external coordinates, only the pair with the highest mapping quality was retained.

### SNP identification and genotyping

SNP calling was performed for parents and progenies using SAMtools software [49]. Then, a Perl script was used to filter the SNPs that had more than two genotypes. Polymorphic markers between the two parents were detected and classified into eight segregation patterns (ab × cd, ab × cc, cc × ab, ef × eg, hk × hk, nn × np, lm × ll, and aa × bb) according to the CP model in JoinMap 4.0 software [53]. After the parental markers were developed, the 116 progeny lines were genotyped for the loci at which the parents differed.

### Linkage map construction

Markers indicating significantly distorted segregation (*P* < 0.001), integrity (> 65%), or containing abnormal bases were filtered by JoinMap 4.0 (JoinMap® 4.0: Software for the calculation of genetic linkage maps in experimental populations). The segregation patterns hk × hk and nn × np were used for the construction of the male parent map, while the patterns lm × ll and hk × hk were used for the female parent map using JoinMap 4.0. The regression algorithm, three times circulation sequence, and Kosambi mapping functions were used in marker distance calculation [54]. The LOD value was set to 2.0–10. The integrated map for both the male and female parents was computed using the combined group for map integration function in MergeMap software [55]. A Perl script SVG was used to visualize the exported maps, and heat maps were constructed to evaluate the maps.

### QTL analysis

QTLs were detected using the software MapQTL (6.0) [56]. Multiple QTL mapping (MQM) was applied to map the QTLs and estimate their effects. The LOD score of the significant QTLs was determined by conducting test analyses (a permutation test with 1000 permutations). However, when this was done, very few QTLs were detected. We thus used LOD = 3.0 for the further analyses.

## Additional files

Additional file 1: **Table S1.** Number of clean GBS reads per sample (XLSX 12 kb)

Additional file 2: **Table S2.** Total number and heterozygosis SNP rate per sample. (XLSX 14 kb)

Additional file 3: **Table S3.** Genetic linkage group statistics of the male map (XLSX 11 kb)

Additional file 4: **Table S4.** Genetic linkage group statistics of the female map (XLSX 12 kb)

Additional file 5: **Figure S1.** Heat map of the group 1 of the male map (A), female map (B) and integrate map (C). The x-axis and y-axis are the names of the markers. The color is correlated with linkage strength. (DOCX 130 kb)

Additional file 6: **Table S5.** QTL locations. (XLSX 19 kb)

## Abbreviations

BT: Bifurcate trunk
CW: Crown width
GBS: Genotyping by sequencing
GD: Total genetic distance of chromosomes
H1: Seedling height of 1 year
H3: Seedling height of 3 year
HBT: The height of bifurcate trunk
HGR: Height growth rate per year
LL: Leaf length
LW: Leaf width
MAS: Marker Assisted Selection
MBA: Maximum branching angle
MBD: Maximum branching diameter
PSN: Primary shoot numbers
PVE: Percentage of phenotypic variation explained by each QTL
QTL: Quantitative trait loci
SBD1: Stem base diameter of 1 year
SBD3: Stem base diameter of 3 year
SBDGR: SBD growth rate per year
SEBLFs: Subtropical evergreen broad-leaved forests
