# Supplementary material for: A high-density genetic map of Schima superba based on its chromosomal characteristics
Source: BMC Plant Biol. 2019 Jan 25;19:41. doi: 10.1186/s12870-019-1655-8 (PMC6347745; doi:10.1186/s12870-019-1655-8)
Supplement: Supplementary file 5 — Figure S1. Heat map of the group 1 of the male map (A), female map (B) and integrate map (C). The x-axis and y-axis are the names of the markers. The color is correlated with linkage strength. (DOCX 130 kb) [file 12870_2019_1655_MOESM5_ESM.docx]

| 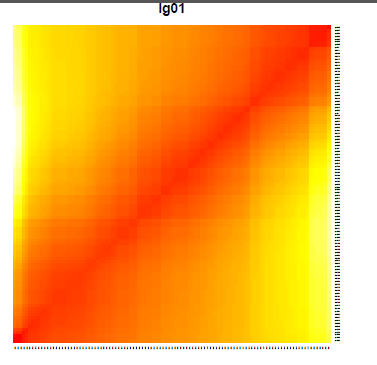 | 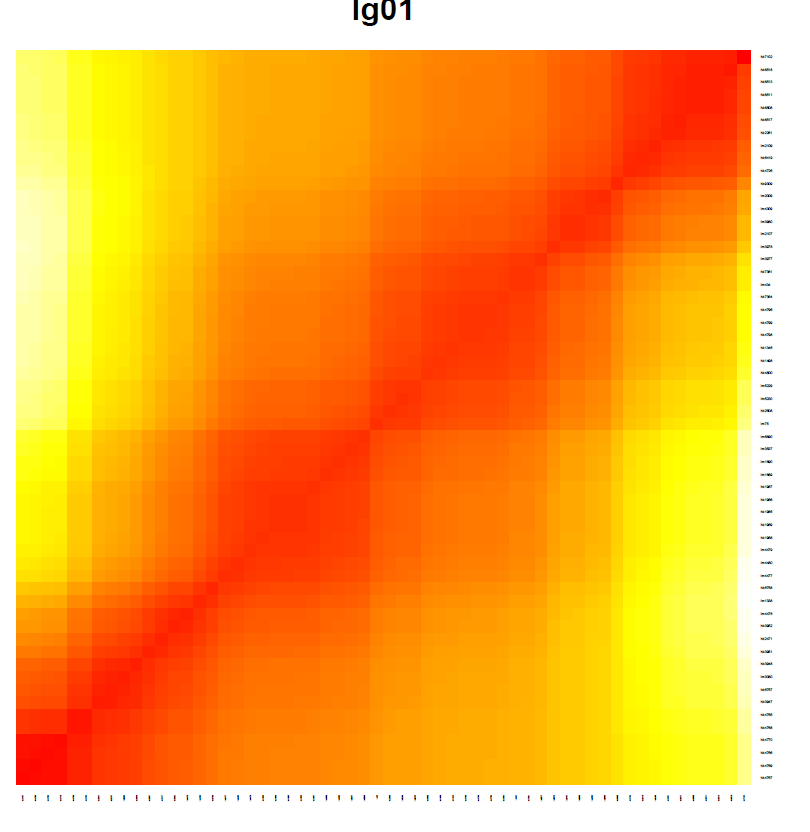 |
| --- | --- |
| A | B |
| 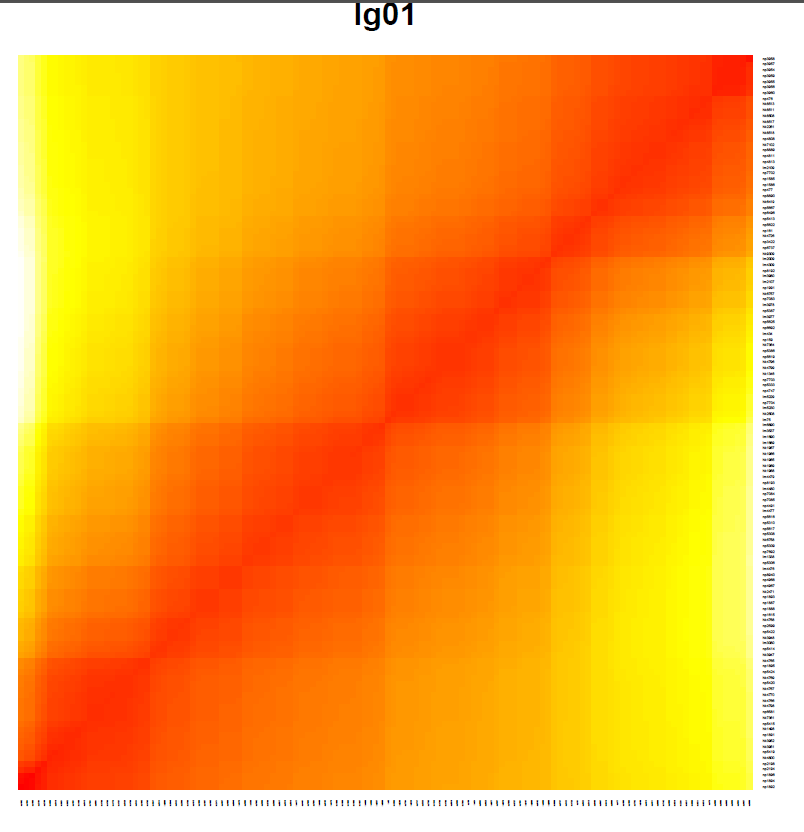 |  |
| C |  |

Additional file 4: Figure S1 Heat map of the group 1 of the male map (A), female map (B) and integrate map (C). The *x*-axis and *y*-axis are the names of the markers. The color is correlated with linkage strength.
